# Risk Factors for Possible Dementia Using the Hopkins Verbal Learning Test and the Mini-Mental State Examination in Shanghai

**DOI:** 10.3390/diagnostics5040487

**Published:** 2015-11-23

**Authors:** Xin Xu, Shifu Xiao, Tri Budi Rahardjo, Eef Hogervorst

**Affiliations:** 1 Memory, Ageing and Cognition Centre, National University Health System, Singapore 117600, Singapore; 2 Department of Geriatric Psychiatry, Shanghai Mental Health Centre, Shanghai Jiaotong University School of Medicine, Shanghai 200030, China; 3 Centre for Aging Studies, Universitas Indonesia, Depok, Jawa Barat 16424, Indonesia; E-Mail: tri.budi.wr@gmail.com; 4 Psychology Division, School of Sport, Exercise and Health Sciences, Brockington Building, Ashby Road, Loughborough University, Loughborough LE11 3TU, UK

**Keywords:** prevalence, dementia, risk factors

## Abstract

Using a combination of the Hopkins Verbal Learning Test (HVLT) and the Mini-Mental State Examination (MMSE), we investigated the prevalence of possible dementia (DEM) in community-dwelling elderly in Shanghai. Subsequently, we investigated significant risk factors for DEM and generated a DEM self-checklist for early DEM detection and case management. We found that among a total of 521 participants using a HVLT cut-off score of <19 and a MMSE cut-off score of <24, a total of 69 DEM cases were identified. Risk factors, such as advanced age (≥68 years), low education (no or primary level), self-reported history of hypertension, and self-reported subjective memory complaints (SMC) were significantly predictive of DEM. The presence of ≥3 out of four of the above mentioned risk factors can effectively discriminate DEM cases from non-DEM subjects.

## 1. Introduction

Accompanying a rapidly aging population in China, dementia has growing importance. As a progressive degenerative disorder that causes declines in memory, intellect, personality, and communication skills [1], dementia has a significant impact on the quality of life. Zhang [2] reported a percentage of 4.6% of dementia in people over 65 years of age in Shanghai. A similar prevalence of 5.6% of dementia cases in rural China was reported using Diagnostic and Statistical Manual of Mental Disorders (DSM-IV) criteria [3]. Zhang [4] also examined dementia subtypes in China, reporting a prevalence of 4.8% for Alzheimer’s disease (AD, the most common form of dementia) and 1.1% for vascular dementia (VaD). These Chinese data are comparable with dementia figures of Western countries. Currently five million Chinese elderly are estimated to be afflicted with dementia. With an estimated 400 million Chinese people over 60 years of age in the next decades and an estimated five percent prevalence of dementia, this would result in one million new cases every year. With an older age being a risk factor for dementia and an ageing population worldwide, dementia will increase globally, especially in the Chinese community, with an expected increase over 300% in dementia cases in the next decades [4].

The clinical diagnosis of dementia is based on neuropsychological testing, medical history and examination to rule out systemic, psychiatric, neurological, and other causes of cognitive impairment, and to identify the pattern of progression [5,6]. However, most clinical screening tools originate from developed countries and do not take into account some of the issues pertaining to many developing countries such as:

(i) a general lack of resources (e.g., a lack of trained staff, time, and financial constraints); and
(ii) high rates of illiteracy and cultural/linguistic differences which can affect the validity of neuropsychological tests.

Within this context, using brief cognitive tests with excellent discriminant ability is essential for early detection of individuals at high risk of dementia as it allows early management of patients before dementia actually occurs.

Together with the Mini-Mental State Examination (MMSE) which is a commonly used dementia screening tool, the Hopkins Verbal Learning Test (HVLT) has been frequently used to conduct dementia and Mild Cognitive Impairment (MCI) screening in clinical and community-based settings [6,7,8,9,10]. Adding on to its excellent dementia screening capacity, the HVLT can be used as part of treatment trials and is less influenced by demographic factors, such as age and education, compared to the MMSE. Furthermore, the HVLT has been translated in multiple languages and it has been proven feasible to administer it in different countries with minimal impact from culture/ethnicity [11].

In the present study, we aimed to investigate the prevalence of possible dementia (DEM) using the HVLT and the MMSE. Subsequently we investigated DEM risk factors to facilitate DEM self-assessment in community-dwelling elderly in Shanghai.

## 2. Experimental Section

The present cross-sectional study was carried out between June and August, 2011. A total of 521 participants aged between 50 and 95 were recruited from a well-defined community in Shanghai [10]. Ethical approval was obtained from the Shanghai Mental Health Centre before the study was initiated (study reference no. 2012-19).

All participants underwent a comprehensive cognitive assessment covering tests on the following cognitive domains—attention, language, verbal memory (Verbal Paired-Associate Learning), visual memory, and executive functioning, followed by a standardized clinical history examination covering hypertension, hyperlipidemia, diabetes, ischemic heart disease and smoking. All subjects and caregivers were asked the question “Do you think you have problems with your memory”/“Do you think the subject has problems with his/her memory” in order to collect information on subjective memory complaints (SMC). The presence of SMC was defined by at least one “yes” to either question.

Subsequently all participants were administered the HVLT (immediate recall only) and the MMSE by experienced psychologists and research assistants blinded to their other cognitive test results. For illiterate participants, words on the HVLT were read aloud to the participants with a 1 s interval between words by the examiner. A research consensus meeting was conducted on a weekly basis among neurologists, clinicians, psychologist, and research personnel to obtain a gold standard dementia diagnoses using DSM-IV-R [6] criteria. Among 521 participants over age 50, 406 were normal controls, 82 had Mild Cognitive Impairment (MCI) according to standard criteria (Petersen, 2000), and 33 had dementia using the DSM-IV-R criteria.

### Statistical Analysis

Receiver Operational Characteristics (ROC) analyses was conducted to establish the optimal HVLT and MMSE cut-off scores for dementia. Subsequently, individuals with possible dementia (DEM) were defined as those who had HVLT and MMSE scores below respective cut-off scores. Suspected dementia risk factors investigated were: an advanced age (≥68 years), low education (≤6 years), a self-reported (and carer-confirmed) history of hypertension, hyperlipidemia, ischemic heart disease, diabetes and smoking and these were entered into logistic regression model simultaneously together with the presence of subjective memory complaints (SMC) to examine the independent contribution of the presence of each variable to DEM. All predictors were binary variables (present or not). A summation risk score was then generated using all significant DEM predictors and ROC was employed to establish the optimal cut-off score for the DEM risk score. All analyses were performed using SPSS 23.0. (IBM, Armonk, NY, USA), using a *p* value of ≤0.05 for significance.

## 3. Results

Study participant’s descriptive analyses are presented in Table 1. Patients with dementia were 10 times more likely to have a history of hypertension. They were on average 14 years older and times more likely to have had low education. Whereas the other vascular risk factors were also different between groups, they were not maintained in logistic regression analyses (see below).

**Table 1:** Descriptive characteristics of study participants.

Characteristics	Whole Sample (*n* = 521)	NCI (*n* = 406)	MCI (*n* = 82)	Dementia (*n* = 33)	*p* Value
Age (years, mean ± SD)	67.5 ± 10.3	65.7 ± 9.7	71.3 ± 10.2	79.8 ± 6.0	<0.001
Education (below Primary School level %)	162 (31.1%)	93 (22.9%)	38 (46.3%)	31 (93.9%)	<0.001
Gender (male %)	237 (45.5%)	191 (47.0%)	35 (42.7%)	11 (33.3%)	NS
Occupation (no job or manual %)	348 (66.8%)	267 (65.8%)	48 (58.5%)	33 (100%)	<0.001
History of Hypertension (yes %)	119 (22.8%)	38 (9.4%)	51 (62.2%)	30 (90.9%)	<0.001
History of Hyperlipidemia (yes %)	47 (9.0%)	15 (3.7%)	25 (30.5%)	7 (21.2%)	<0.001
History of Diabetes (yes %)	33 (6.3%)	10 (2.5%)	20 (24.2%)	3 (9.1%)	<0.001
History of Ischemic Heart Disease (yes %)	67 (12.9%)	22 (5.4%)	34 (41.5%)	11 (33.3%)	<0.001
Subjective Memory Complaint	172 (33.0%)	66 (16.3%)	76 (92.7%)	30 (90.9%)	<0.001
Smoking History (yes %)	129 (24.8%)	103 (25.4%)	17 (20.7%)	9 (27.3%)	NS
MMSE Total Score	26.6 ± 5.2	28.2 ± 3.2	24.5 ± 3.4	12.9 ± 6.6	<0.001
HVLT Total Score	22.4 ± 9.0	25.4 ± 7.1	13.8 ± 5.6	6.8 ± 6.1	<0.001

NCI, No Cognitive Impairment; MCI, Mild Cognitive Impairment; NS, not significant.

**Figure 1:**
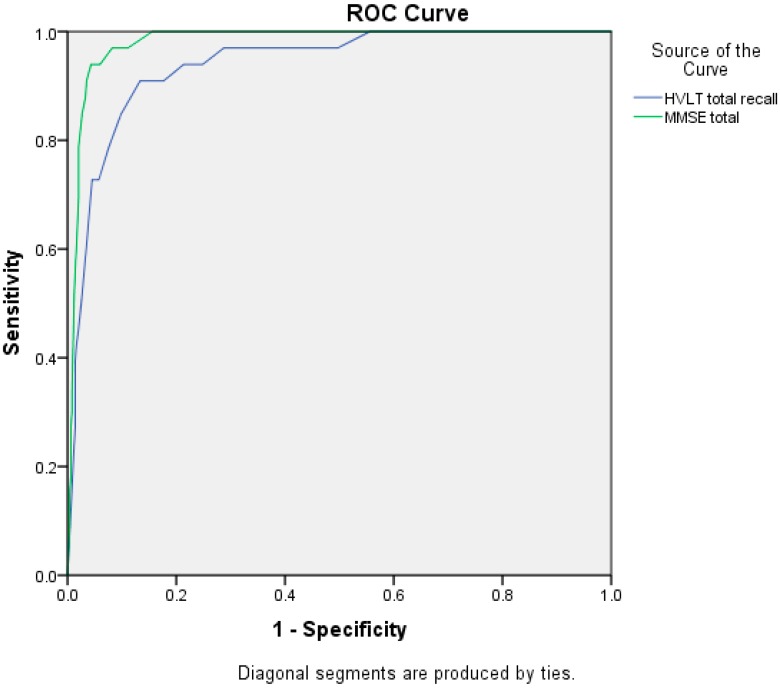
ROC analysis for the HVLT and the MMSE in discriminating dementia patients from non-dementia subjects.

By applying ROC analysis, the optimal HVLT and MMSE cut-off scores were obtained in differentiating dementia patients from non-dementia subjects (Figure 1).

Using an optimal HVLT cut-off of <19 and a MMSE cut-off of <24 (Table 2), a total of 69 subjects were included DEM group and 452 were in non-DEM group.

**Table 2:** Area Under Curves (AUCs), optimal cut-off scores, sensitivity (SE) and specificity (SP) for the HVLT and the MMSE in discriminating dementia patient from non-dementia subjects.

Tests	AUC (95%CI)	Cut-Off Score	SE	SP
HVLT	0.94 (0.90–0.98)	<19 *	0.97	0.87
		<14	0.91	0.89
MMSE	0.98 (0.97–0.99)	<24 *	0.97	0.92
		<22	0.94	0.96

* Optimal cut-off.

Table 3 shows the distribution of DEM and non-DEM participants in their original clinical consensus diagnostic groups. Data shows that 67.1% of MCI patients and 93.9% of dementia patients were in the DEM group whilst 99.5% of NCI controls are in the non-DEM group (*p* < 0.001).

**Table 3:** Distribution of DEM and non-DEM participants in clinical consensus diagnostic groups.

Groups	DEM (*n* = 69)	Non-DEM (*n* = 452)	*p* Value
Controls (*n* = 406)	1 (0.2%)	404 (99.5%)	<0.001
MCI (*n* = 82)	55 (67.1%)	27 (32.9%)	
Dementia (*n* = 33)	31 (93.9%)	2 (6.1%)	

DEM, possible dementia; MCI, Mild Cognitive Impairment.

Logistic regression models using these cut-offs showed that among all dementia risk factors, an age of ≥68 years, a lower education (of no or only obtained primary level *versus* more), history of hypertension, and the presence of SMC were significant independent DEM predictors (Table 4).

**Table 4:** Logistic regression analyses for dementia risk factors in predicting possible dementia (DEM).

Dementia Predictors	Odds Ratio (OR)	95% CI	*p* Value
Advanced Age (≥68 years)	4.5	1.8–11.2	0.001
Low Education (No or Primary Level)	7.9	3.5–17.8	<0.001
Gender (Female)	2.4	0.9–6.7	NS
History of Hypertension (Yes)	4.9	2.0–11.7	<0.001
History of Hyperlipidemia (Yes)	1.4	0.5–3.8	NS
History of Diabetes (Yes)	0.7	0.2–2.1	NS
History of Ischemic Heart Disease (Yes)	0.6	0.5–2.9	NS
History of Smoking (Yes)	0.4	0.2–2.0	NS
Subjective Memory Complaint (Yes)	8.3	3.2–21.8	<0.001

NS, not significant.

Subsequently a four-point ordinal DEM risk scale was generated using these significant predictors. One point was awarded when a significant predictor was present.

**Figure 2:**
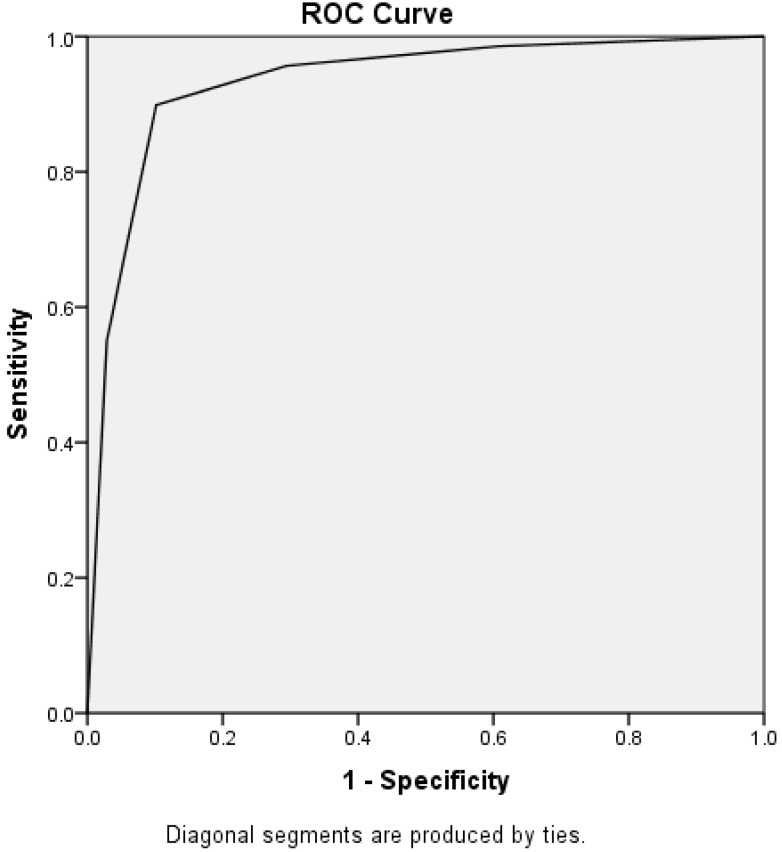
ROC analysis for DEM risk scale in discriminating DEM cases from non-DEM subjects.

ROC analysis showed that the DEM risk scale obtained an AUC of 0.93 (95% CI = 0.90–0.97) (Figure 2). A DEM risk score of ≥3 provided optimal discriminatory capacity in differentiating DEM subjects from normal controls, rendering 90% for both sensitivity (SE) and specificity (SP).

## 4. Discussion

The main finding of the present study is that by using a combination of HVLT and MMSE cut-off scores, community-dwelling elderly at high risk of possible dementia can be identified in Shanghai. Furthermore, we generated a useful possible dementia risk scale for individuals to assess their risk of developing dementia and found that 90% individuals scoring three and above on this scale were in the possible dementia group.

It is worth mentioning that the optimal cut-off scores for the HVLT and the MMSE are consistent with previous studies whereby a HVLT score of 18/19 and a MMSE score of 23/24 could effectively differentiate dementia cases from non-dementia subjects in both Western and Asian settings [7,10]. This further confirms that the combination of optimal HVLT and MMSE cut-off scores can be applied in different countries with minimal further modification for ethnic/cultural differences. Previous work in Beijing [9] suggested that using different age modified cut-off categories might further improve specificity (e.g., for young-old <65, using the cut-off of 18/19 and for the older old 65–80, a cut-off of 14/15 on the total recall of the HVLT).

**Figure 3:**
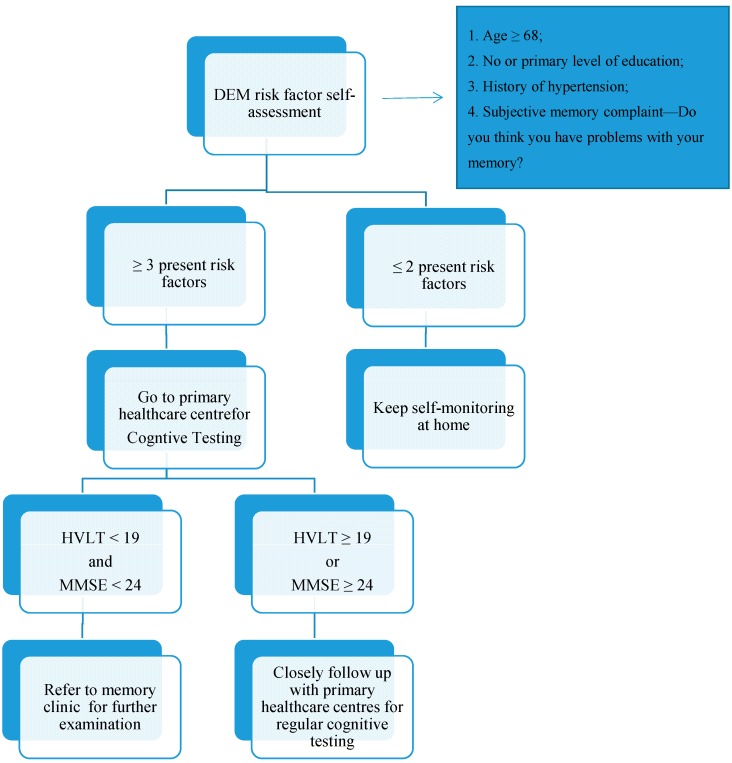
Proposed DEM self-checklist and case management flowchart.

With cognitive screening tests and DEM risk factors, we proposed a DEM self-checklist and case management flowchart (Figure 3).

Dementia is a relatively common disease in those over 65 years of age which prevalence could be underestimated in community-dwelling elderly. In the present study, a total of 69 elderly were found to be at high risk of having dementia using a combination of the HVLT and the MMSE, among whom, however, only 31 had previously been diagnosed with dementia, which composes less than 50% of the high risk population. In terms of MCI patients in the present study, 67.1% (55 out of 82) were classified as DEM cases. It is widely acknowledged that not all MCI cases convert to dementia at a later stage. Hence it is important to identify those who are more likely to develop early dementia. The combination of the HVLT and the MMSE can help distinguish between these groups. These MCI cases are in need for a follow-up to see if they develop a clearer dementia picture. Dementia is associated with dependency which indicates a state of high vulnerability preceding the onset of overt disability and high care needs. After investigating key risk factors for possible dementia, it is mandatory to develop an intervention program for elderly subjects at high risk and reduce the risk for morbidity and disability. Several previous studies have reported that midlife hypertension is a significant risk factor for dementia [12,13]. However, antihypertensive medication has overall not been successful in reducing dementia symptoms [14]. Over-medication can also lead to orthostatic hypotension and falls, a risk factor for dependency by itself [15].

Another way of reducing hypertension and increasing stability and muscular strength is by engaging in exercise which can also directly affect brain function and integrity, which was even shown to occur in older animal studies employing exercise programs [16,17]. Exercise has been implicated as effective for improving cognitive function in older adults, yet the results are inconsistent as no overall cognitive improvement was reported in a recent review [18] which suggests that other moderating factors are involved in this intervention process. Other studies did highlight the protective effect of regular physical activity in reducing the risk of cognitive impairment and dementia. Most prospective intervention studies of physical exercise and cognition focused on aerobic-based exercise training [19,20,21]. It was suggested that long-term moderate-intensity aerobic exercise can reliably reverse age-related cognitive impairment. The effect of aerobic exercise training was considered to be on central—rather than on peripheral—function by boosting increased cerebral metabolic activity [22]. However, adherence to these exercises can be low, limiting its effectiveness [18]. Resistance exercise may also improve memory and physical strength with good adherence, which in our studies ranged from 55% (in older institutionalized elderly) to 84% in middle-aged participants [23,24]. Its effect on our DEM risk scale needs to be investigated in more detail to see if it can reliably reduce blood pressure and other risk factors.

In recent years, researchers have raised awareness on SMC in normal controls. It has been reported that the presence of SMC, even in the absence of objective memory deficits, may predict subsequent cognitive decline and dementia [25,26,27]. These studies suggest that a better focus on SMC may help identify individuals at high risk of developing cognitive dysfunction and possible dementia.

The main limitation to the present study is that dementia group had a relative small sample size (6.3% among the whole sample) with advanced age (97.0% were older than 68 years of age) and low levels of education (93.9% had obtained less or equal than six years). This limited subsequent analysis for different age and education strata.

## 5. Conclusions

In conclusion, we found that a combination of the HVLT and the MMSE can be used to detect possible dementia. Longitudinal studies are required to establish the prognostic validity of the combined measurement for disease progression. Previous work by Schrijnemaekers [28] showed the validity of MCI and dementia progression using the HVLT and MMSE, which improved in controls and showed a progressive decline in dementia over a 2–3 year follow-up. This work needs to be extended to Asia. By combing the measurement of cognitive testing and possible dementia risk factors, early detection and case management of dementia possibly by using exercise studies can be effectively applied.
